# Supplementary material for: Avoidance of carnivore carcasses by vertebrate scavengers enables colonization by a diverse community of carrion insects
Source: PLoS One. 2019 Aug 29;14(8):e0221890. doi: 10.1371/journal.pone.0221890 (PMC6715269; doi:10.1371/journal.pone.0221890)
Supplement: S1 Table — (DOCX) [file pone.0221890.s001.DOCX]

**S1 Table.** **GPS coordinates of carcasses.**

| Study area | Carcass | Latitude | Longitude |  |
| --- | --- | --- | --- | --- |
| Espuña | ES-01 | 37.865521 | -1.5293075 | |
|  | ES-02 | 37.860481 | -1.5449583 | |
|  | ES-03 | 37.860543 | -1.5591192 | |
|  | ES-04 | 37.856813 | -1.5743839 | |
|  | ES-05 | 37.863406 | -1.5850312 | |
|  | ES-06 | 37.871078 | -1.5720376 | |
|  | ES-07 | 37.871480 | -1.5781915 | |
|  | ES-08 | 37.852038 | -1.5643143 | |
|  | ES-09 | 37.854057 | -1.5449523 | |
|  | ES-10 | 37.856239 | -1.5278427 | |
| Bebedor | BE-01 | 38.141589 | -2.0571849 | |
|  | BE-02 | 38.145067 | -2.0412905 | |
|  | BE-03 | 38.139458 | -2.0335369 | |
|  | BE-04 | 38.136972 | -2.0411403 | |
|  | BE-05 | 38.147553 | -2.0327397 | |
|  | BE-06 | 38.149495 | -2.0239985 | |
|  | BE-07 | 38.146474 | -2.0061436 | |
|  | BE-08 | 38.133627 | -2.0155599 | |
|  | BE-09 | 38.131112 | -2.0352584 | |
|  | BE-10 | 38.128316 | -2.0451598 | |
