# Supplementary material for: Avoidance of carnivore carcasses by vertebrate scavengers enables colonization by a diverse community of carrion insects
Source: PLoS One. 2019 Aug 29;14(8):e0221890. doi: 10.1371/journal.pone.0221890 (PMC6715269; doi:10.1371/journal.pone.0221890)
Supplement: S2 Table — (DOCX) [file pone.0221890.s002.DOCX]

**S2 Table.** **List of insect taxa collected during each visit in the two study areas.** A: adult; E: eggs; L_:_ larva (for Diptera larvae, L1, L2 and L3 indicates first-, second- and third-instars, respectively). For each visit, the number of carcasses where each taxon was collected is provided.

| Study area | Order | Family | Species | Stage | No. of carcasses visited by a given taxon | | | |
| --- | --- | --- | --- | --- | --- | --- | --- | --- |
| Espuña |  |  |  |  | 1^st^ visit | 2^nd^ visit | 3^rd^ visit | 4^th^ visit |
|  | Diptera | Calliphoridae | *Calliphora* sp. | E | 10 | 10 | 2 | 0 |
|  |  |  | *Calliphora vicina* Robineau-Desvoidy, 1830 | A | 10 | 10 | 4 | 2 |
|  |  |  |  | L1 | 0 | 6 | 0 | 0 |
|  |  |  |  | L2 | 0 | 6 | 5 | 0 |
|  |  |  |  | L3 | 0 | 0 | 7 | 1 |
|  |  |  | *Calliphora vomitoria* (Linnaeus, 1758) | A | 9 | 10 | 5 | 2 |
|  |  |  |  | L1 | 0 | 3 | 4 | 0 |
|  |  |  |  | L2 | 0 | 0 | 4 | 1 |
|  |  |  |  | L3 | 0 | 0 | 7 | 4 |
|  |  | Heleomyzidae | *Tephroclamys* sp. | A | 0 | 1 | 0 | 0 |
|  |  | Muscidae | *Muscina levida* (Harris, 1780) | A | 2 | 0 | 1 | 0 |
|  |  | Phoridae | *Megaselia* sp. | A | 1 | 0 | 0 | 0 |
|  |  | Piophilidae | *Prochyliza nigrimana* (Meigen, 1826) | A | 0 | 4 | 1 | 0 |
|  |  | Sciaridae | Unidentified species | A | 1 | 1 | 0 | 0 |
|  |  | Sphaeroceridae | *Coproica* sp. | A | 0 | 0 | 1 | 0 |
|  |  |  | *Leptocera* sp. | A | 3 | 4 | 3 | 4 |
|  | Coleoptera | Cleridae | *Necrobia violacea* (Linnaeus, 1758) | A | 0 | 0 | 3 | 1 |
|  |  | Dermestidae | *Dermestes frischii* Kugelann, 1792 | A | 0 | 0 | 5 | 8 |
|  |  |  |  | L | 0 | 0 | 5 | 9 |
|  |  |  | *Dermestes hankae* Háva, 1999 | A | 0 | 0 | 2 | 0 |
|  |  |  | *Dermestes undulatus* Brahm, 1790 | A | 0 | 0 | 1 | 7 |
|  |  |  |  | L | 0 | 0 | 0 | 3 |
|  |  | Histeridae | *Saprinus detersus* (Illiger, 1807) | A | 0 | 2 | 4 | 5 |
|  |  |  | *Saprinus furvus* Erichson, 1834 | A | 0 | 0 | 1 | 0 |
|  |  | Nitidulidae | *Nitidula flavomaculata* Rossi, 1790 | A | 0 | 0 | 1 | 2 |
|  |  | Silphidae | *Thanatophilus ruficornis* (Kuster, 1851) | A | 0 | 0 | 4 | 3 |
|  |  |  |  | L | 0 | 0 | 0 | 3 |
|  |  |  | *Thanatophilus rugosus* (Linnaeus, 1758) | A | 0 | 0 | 3 | 1 |
|  |  | Staphylinidae | *Creophilus maxillosus* (Linnaeus, 1758) | A | 0 | 0 | 1 | 2 |
|  |  |  | Aleocharinae sp. | A | 7 | 10 | 7 | 7 |
|  |  | Trogidae | *Trox perlatus hispanicus* Harold, 1872 | A | 0 | 2 | 0 | 2 |
|  | Hymenoptera | Braconidae | *Alysia manducator* (Panzer, 1799) | A | 2 | 6 | 0 | 0 |
|  |  | Formicidae | Unidentified species | A | 2 | 0 | 0 | 3 |
|  |  | Vespidae | *Vespula vulgaris* (Linnaeus, 1758) | A | 8 | 7 | 1 | 0 |
| Bebedor |  |  |  |  | 1^st^ visit | 2^nd^ visit | 3^rd^ visit | 4^th^ visit |
|  | Diptera | Calliphoridae | *Calliphora* sp. | E | 10 | 10 | 1 | 0 |
|  |  |  | *Calliphora vicina* Robineau-Desvoidy, 1830 | A | 7 | 1 | 4 | 2 |
|  |  |  |  | L1 | 0 | 5 | 0 | 0 |
|  |  |  |  | L2 | 0 | 6 | 1 | 0 |
|  |  |  |  | L3 | 0 | 4 | 3 | 0 |
|  |  |  | *Calliphora vomitoria* (Linnaeus, 1758) | A | 6 | 1 | 4 | 2 |
|  |  |  |  | L1 | 0 | 3 | 0 | 0 |
|  |  |  |  | L2 | 0 | 3 | 0 | 0 |
|  |  |  |  | L3 | 0 | 2 | 9 | 0 |
|  |  |  | *Chrysomya albiceps* (Wiedemann, 1819) | A | 1 | 0 | 0 | 0 |
|  |  | Fanniidae | *Fannia canicularis* (Linnaeus, 1761) | A | 2 | 0 | 0 | 0 |
|  |  | Muscidae | *Muscina levida* (Harris, 1780) | A | 5 | 3 | 0 | 0 |
|  |  | Piophilidae | *Prochyliza nigrimana* (Meigen, 1826) | A | 1 | 0 | 0 | 0 |
|  |  | Sarcophagidae | *Sarcophaga crassipalpis* Macquart, 1839 | A | 2 | 0 | 0 | 0 |
|  | Coleoptera | Cleridae | *Necrobia violacea* (Linnaeus, 1758) | A | 0 | 0 | 3 | 2 |
|  |  | Dermestidae | *Dermestes frischii* Kugelann, 1792 | A | 0 | 3 | 3 | 5 |
|  |  |  |  | L |  | 3 | 5 | 6 |
|  |  |  | *Dermestes undulatus* Brahm, 1790 | A | 0 | 0 | 0 | 3 |
|  |  |  |  | L | 0 | 0 | 0 | 0 |
|  |  | Histeridae | *Saprinus detersus* (Illiger, 1807) | A | 0 | 6 | 4 | 4 |
|  |  |  | *Saprinus furvus* Erichson, 1834 | A | 0 | 0 | 1 | 0 |
|  |  | Nitidulidae | *Nitidula flavomaculata* Rossi, 1790 | A | 0 | 3 | 0 | 3 |
|  |  | Silphidae | *Thanatophilus ruficornis* (Kuster, 1851) | A | 0 | 7 | 5 | 1 |
|  |  |  |  | L | 0 | 0 | 7 | 8 |
|  |  |  | *Thanatophilus rugosus* (Linnaeus, 1758) | A | 0 | 0 | 3 | 1 |
|  |  | Staphylinidae | *Creophilus maxillosus* (Linnaeus, 1758) | A | 0 | 2 | 2 | 0 |
|  |  |  | Aleocharinae sp. | A | 0 | 1 | 1 | 1 |
|  |  | Trogidae | *Trox perlatus hispanicus* Harold, 1872 | A | 0 | 2 | 3 | 6 |
|  | Hymenoptera | Braconidae | *Alysia manducator* (Panzer, 1799) | A | 3 | 6 | 0 | 0 |
|  |  | Formicidae | Unidentified species (A) | A | 2 | 1 | 0 | 1 |
|  |  | Vespidae | *Vespula vulgaris* (Linnaeus, 1758) | A | 3 | 1 | 0 | 0 |
