# Supplementary material for: Avoidance of carnivore carcasses by vertebrate scavengers enables colonization by a diverse community of carrion insects
Source: PLoS One. 2019 Aug 29;14(8):e0221890. doi: 10.1371/journal.pone.0221890 (PMC6715269; doi:10.1371/journal.pone.0221890)
Supplement: Attachment — Submitted filename: Munoz-Lozano_et_al_MS_Response_to_Reviewers.docx [file pone.0221890.s003.docx]

Dear Editor,

We are submitting a revised version of the manuscript entitled “Avoidance of carnivore carcasses by vertebrate scavengers enables colonization by a diverse community of carrion insects” (manuscript ID PONE-D-19-16398), modified in accordance with the comments and suggestions from the reviewers. The changes made in the manuscript are detailed below, together with the responses to the correspondent reviewer’s comments. We are grateful to the two reviewers for their helpful and constructive recommendations and overall positive feedback, and we hope that the manuscript can be accepted for publication in PLOS ONE.

Yours sincerely,

Daniel Martín-Vega

Reviewers' comments:

Reviewer #1:

Dear Editor, the paper PONE-D-19-16398 by Munoz-Lozano and co-workers deals with a very important topic in forensic entomology especially when wildlife is considered.

experimental design, number of replicates and the statisics applied are sound and very well developed.

Only few minor observations to improve the text:

General

Add the Authority and the year of description of all the species (vertebrates as well) when reported the first time

*Our response: The authority and year of description have been added for all the species when mentioned in the main text for the first time, as well as on supplementary Table S2 (“Table S1” in the previous version of the manuscript).

Introduction

-add a sentence indicating that as well some work on microbiome of wild animals has been performed (eg.: Tuccia F, Zurgani E, Bortolini S, Vanin S Experimental evaluation on the applicability of necrobiome analysis in forensic veterinary science. Microbiologyopen. 2019 Mar 12:e828. doi: 10.1002/mbo3.828)

*Our response: The following sentence with the suggested reference has been included: “In a similar way, some studies have also explored the potential of the changes in the bacterial communities during the decomposition process of animal carcasses as indicators for death time estimations in forensic veterinary science [23]” (lines 43–45 of the new version of the manuscript).

line 37, mention as well the very important book published by M. Eric Benbow, Jeffery K. Tomberlin, Aaron M. Tarone in 2018 (Carrion Ecology, Evolution, and Their Applications)

*Our response: The suggested reference has been included (new reference number [17]) on line 37, as well as the reference of a book recently published which is also relevant to the present paper (Olea PP, Mateo-Tomás P, Sánchez-Zapata JA, editors. Carrion Ecology and Management. Dordrecht: Springer, 2019; new reference number [18]). All the reference numbers have been revised and updated accordingly in the new version of the manuscript.

line 38 add "and veterinary" after medicolegal

*Our response: Added as suggested.

line 51 add "or coancealed" after [2]

*Our response: Added as suggested (line 54 of the new version of the manuscript).

line 55 affect may be better than condition

*Our response: “condition” has been changed to “affect” (line 58 of the new version of the manuscript)

line 58 add "being" at the end of the line

*Our response: Added as suggested (line 62 of the new version of the manuscript).

line 62 delete "Forensic" at the beginning of the sentence

*Our response: “Forensic” has been deleted as suggested (line 65 of the new version of the manuscript).

line 63 add "of" after surrogate

*Our response: Added as suggested (line 66 of the new version of the manuscript).

M&M

-add a space between the degree symbol and C

*Our response: Thank you for this suggestion. We have checked style manuals and several previous papers published in PLOS ONE and we have found that the number, degree symbol and capital C are written together. We have left it in this way; nevertheless, we will be happy to change it if another way is preferred by the journal.

line 100 add as well the max and minimum temperature

*Our response: Maximum and minimum temperatures have been added in lines 113–115 and 117–119 of the new version of the manuscript.

line 131 bette 600 m than 0.6 Km...in the whole paragraph you are using m as unit

*Our response: Changed as requested (line 157 of the new version of the manuscript).

lines 200 and 203 check the way in which the references are cited (apex)

*Our response: Reference style has been amended and the reference number updated (reference 61 in line 226 and reference 620in line 229 of the new version of the manuscript).

Results

line 272 vulgaris not vularis

*Our response: The species name has been corrected (line 305 of the new version of the manuscript).

line 352 change similar with related

*Our response: Changed as suggested (line 386 of the new version of the manuscript).

line429 add to the reference 67 as well Bugelli et al., Decomposition pattern and insect colonization in two cases of suicide by hanging Forensic Sci Res. 2018; 3(1): 94–102. doi: 10.1080/20961790.2017.1418622

*Our response: The suggested reference (reference [71]) has been added (line 464 of the new version of the manuscript).

Reviewer #2:

It is an interesting manuscript related with carrion ecology. Contribution is original and worthy of publication in PLoS ONE. The authors discuss about most of the weaknesses of experiment. I would like to suggest some additional corrections.

1. Could provide (maybe in appendix) detailed data (GPS coordinates) for particular exposed experimental carrions? Small scale differences in carrion exposition may have very strong impact on insect succession. Sometimes it is stronger than between distant locations but where carrions are exactly in the same habitats and with the same abiotic conditions.

*Our response: we agree with the reviewer that this is an important point that deserves more details. Accordingly, we have included a map of the study areas showing the location of carcasses (see new Fig. 1) and a table with the GPS coordinates of them (see new Table S1). Moreover, we have added some words to the main text to explain that we were very careful in selecting the carcass sites, so the microhabitat surrounding all of them was similar (“Carcasses were randomly placed in southern-oriented sites with intermediate vegetation cover [42]”; see lines 154-155 of the new version of the ms).

2. Experimental fox carrion was not eaten by vertebrate scavengers (except golden eagle). However authors noticed many incidents of visiting of experimental carrion by many vertebrate species. Could you describe this contact with more details? Has any physical contact with carrion occurred (jerking, moving etc.)?

*Our response: please note that we detected “some”, but not “many”, visits of few vertebrate scavenger species to carcass sites. In particular, cameras recorded 22 photographs of 5 vertebrate scavenger species in one study area (i.e., 2.2 photos/carcass and 1.3 species/carcass as average) and 20 photographs of 3 vertebrate scavenger species in the other study area (i.e., 2 photos/carcass and 0.4 species/carcass as average), as we already mentioned in results (lines 236-237 and 247-248 in the new version). According to the reviewer’s suggestion, we specify in the new version of the ms that “No other vertebrate species were recorded to contact physically with carcasses.” (Espuña study area; lines 245-246) and that “No vertebrate species were recorded to contact physically with carcasses, except a group of four wild boar piglets that probably touched one carcass. In this case, however, the potential contact was not sufficient to move the carcass.” (Bebedor study area; lines 252-255).

3. I have some hesitation related to quite low number of repetitions in the experiment. The information about detailed position of particular experimental carrions (suggested above) may tell us that material from each carrion is really comparable to others.

*Our response: we thank the reviewer for identifying which could be a potential limitation of our study. Getting fox carcasses for this kind of experiments is a difficult task, and that is probably why the scavenging patterns of fox carcasses have hardly been studied to date. Thus, as explained in our response to the first question above, we were very careful in selecting the sites in which we placed carcasses in order to make them comparable within and between study areas. In fact, our GLMM analyses confirmed that carcass identity was not an important variable to explain the variability in our response variables; in contrast, study area and, mostly, visit explained an important part of such variability.

4. I’m not sure (in contrary to authors…) that evisceration of carrion is without serious influence on the insect succession. Did authors do any tests for comparison of insect succession for eviscerated vs. eviscerated fox carrion? If not, it should be mentioned in the text.

*Our response: again, the reviewer highlights a good point. We always avoided exposing the open part of the carcasses to minimize the effect of the evisceration on scavenging patterns. We now specify this in lines 153-154. In any case, we feel that we already were cautious on this regard. As we discussed in the previous version, “It should also be noted that we used necropsied and eviscerated red fox carcasses, in order to minimise the risk of pathogen transmission [41]. The potential effect of the use of eviscerated carcasses on the patterns of carrion consumption by both vertebrate and invertebrate scavengers deserves further study. However, several studies have demonstrated that the rate of decomposition and the pattern of insect colonisation do not differ between intact and necropsied and/or wounded carcasses [75, 76]. In this sense, the composition of the insect fauna recorded during our study was overall similar to that reported from non-eviscerated, small- and medium-sized pig carcasses which were protected from vertebrate scavenging [22, 30]. Furthermore, our experimental carcasses were colonised by blow flies within the first three days after carcass exposure (Fig. 1). This is in line with the early colonisation patterns of carrion during winter, when the arrival of blow flies can sometimes be delayed a few days, in contrast to warmer months, when carrion can be colonised within hours after death [30, 63].” (lines 506-518 in the new version).
